# Amino Group-Driven Adsorption of Sodium p-Perfluorous Nonenoxybenzene Sulfonate in Water by the Modified Graphene Oxide

**DOI:** 10.3390/toxics12050343

**Published:** 2024-05-08

**Authors:** Mengyuan Lu, Yang Liu, Xinning Zheng, Wenjuan Liu, Yang Liu, Jia Bao, Ao Feng, Yueyao Bao, Jiangyong Diao, Hongyang Liu

**Affiliations:** 1 School of Environmental and Chemical Engineering, Shenyang University of Technology, Shenyang 110870, China; lumengyuan2022@163.com (M.L.); fengao1126@163.com (A.F.); 15851929621@163.com (Y.B.); 2 Shenyang Zhenxing Sewage Treatment Co., Ltd., Shenyang 110143, China; 13610815959@163.com; 3 Dalian Xigang District Center for Disease Control and Prevention, Dalian 116021, China; 15941132830@163.com; 4 Shenyang Hoper Group Co., Ltd., Shenyang 110112, China; liuyang@syhoper.com; 5 Shenyang National Laboratory for Materials Science, Institute of Metal Research, Chinese Academy of Sciences, Shenyang 110016, China; jydiao@imr.ac.cn (J.D.); liuhy@imr.ac.cn (H.L.)

**Keywords:** sodium p-perfluorinated nonenoxybenzene sulfonate (OBS), chitosan-modified amino-driven graphene oxide (CS-GO), removal, adsorption mechanism, electrostatic interaction

## Abstract

Sodium p-perfluorous nonenoxybenzene sulfonate (OBS) is one of the key alternatives to perfluoroalkyl substances (PFASs). Its widespread tendency has increased extensive contamination in the aquatic environment. However, the present treatment technology for OBS exhibited insignificant adsorption capacity and long adsorption time. In this study, three proportions (1:5, 3:5, and 10:1) of chitosan-modified amino-driven graphene oxide (CS-GO) were innovated to strengthen the OBS adsorption capacity, compared with graphene oxide (GO) and graphene (GH). Through the characterization of SEM, BET, and FTIR, it was discovered that CS was synthetized on GO surfaces successfully with a low specific surface area. Subsequently, batch single influence factor studies on OBS removal from simulated wastewater were investigated. The optimum removal efficiency of OBS could be achieved up to 95.4% within 2 h when the adsorbent was selected as CS-GO (10:1), the dosage was 2 mg, and the pH was 3. The addition of inorganic ions could promote the adsorption efficiency of OBS. In addition, CS-GO presented the maximum adsorption energy due to additional functional groups of -NH_3_, and electrostatic interaction was the foremost motive for improving the adsorption efficiency of OBS. Moreover, OBS exhibited the fastest diffusion coefficient in the CS-GO-OBS solution, which is consistent with the fitting results of adsorption kinetics.

## 1. Introduction

Perfluoroalkyl substances (PFASs) are a series of artificial fluorinated hydrocarbons consisting of a carbon backbone fully surrounded by fluorine atoms. Recently, C8–C14 PFASs and their sodium and ammonium salts have been listed in the candidate list of regulatory substances in the EU, and normally the use of perfluorooctane sulfonate (PFOS) is included in Annex B of the Stockholm Convention on Persistent Organic Pollutants (POPs) due to its persistence, bioaccumulative ability, and various toxicities [1,2,3,4,5]. Sodium p-perfluorous nonenoxybenzene sulfonate (OBS, C_9_F_17_OC_6_H_4_SO_3_Na) is represented as one of the key alternatives of PFOS in the production of aqueous film-forming foams, photographic films, alcohol-resistant foams, and oil production agents [6,7], owing to its high surface activity, strong physical and chemical stability, and hydrophobic and oleophobic characteristics. 

However, due to the inferior technical performance of OBS, larger quantities of OBS are employed to achieve a similar performance to PFOS. This widespread tendency of OBS has increased extensive attention in the distribution of various environmental media, especially in aquatic environments [8,9,10]. For instance, Xu et al. discovered OBS in an oilfield in northern China in 2017, with a maximum concentration of 3.2 × 10^3^ ng/L [11]. Tang et al. investigated the pollution status of PFAS in Poyang Lake of China in 2022, finding that OBS was the most important PFAS contamination in suspended particulate matter with a concentration four times that of PFOS, and exceeded PFOS in water’s dissolved phase and sediment [12]. Meanwhile, Hou et al. found that the OBS concentrations were all 1–4 times higher than other PFAS contaminations in environmental media and organisms near the fluorochemical plant, including surface water, soil, sediment, and even crucian carp [13]. 

Numerous previous studies have shown that exposure to OBS could lead to biotoxicity and bioaccumulation [14]. For instance, pregnancy in mice exposed to OBS could lead to bioaccumulation in the embryo or placenta even in rather low concentrations [15,16,17]. In addition, scientists have observed that zebrafish exposure to high concentrations of OBS (>25.5 mg/L) presented negative effects on heart development, blood pressure, and lipid metabolism, and even led to death, as well as had worse destructive effects on the thyroid than PFOS [2,18,19]. Moreover, OBS has been discovered in maternal serum and placentas near the fluorochemical plants in Fuxin [20]. Therefore, it is necessary to explore an appropriate technology for the efficient removal of OBS in water bodies.

In general, OBS is difficult to remove through wastewater treatment plants due to high-energy C-F bonds and the stable benzene ring structure of OBS. At present, the degradation treatment technology of OBS mainly focuses on advanced oxidation and catalytic oxidation technology. For instance, the UV/persulfate (UV/PS) system could degrade OBS with quick efficiency, but the defluorination rate was only 27.6%, and a series of complex byproducts might be formed during the process, which increases the additional cost and secondary pollution [21]. Wang et al. investigated the reduced graphene oxide/Fe_3_O_4_/H_2_O_2_ system for the catalytic degradation of OBS, which could almost remove OBS from water completely, whereas, it took up to 48 h for the degradation [22]. 

Adsorption technology is widely used in the treatment of contaminants in waters due to the variety of adsorption materials, easy operation, and low cost [23,24]. From 2005 to 2010, a number of conventional materials such as activated carbon and organic montmorillonite were used to adsorb long-chain PFAS depending on the hydrophobic interaction, which showed effective adsorption performance but had a long adsorption time, with even more than 100 h [25,26]. Subsequently, MOFs were used to adsorb PFOA by the mechanisms of electrostatic interaction and Lewis’s acid/base complexation, and the adsorption capacity was achieved at 782.6 mg/g within 60 min, which significantly shortened the adsorption time and improved adsorption performance [27]. In 2020, Klemes et al. explored the β-cyclodextrin co-organic framework (β-CD-COFs) for the adsorption of PFOS, PFBS, PFHxS, and F53B, reaching the adsorption equilibrium in only 2 min. This phenomenon of quick adsorption could be attributed to the synergistic adsorption interaction, including electrostatic interaction, hydrophobic interaction, π–π interaction, and hydrogen bonding interaction [28]. Based upon the above analysis, the electrostatic interaction between the positive electrical materials and anion PFAS could facilitate the adsorption effectively, and the synergistic interactions of the hydrophobic interactions, π–π interactions, and hydrogen bonding were favorable for the adsorption.

OBS is an emerging PFAS which possesses a similar structure to PFOS-containing C-F chains and a terminal anion group of sulfonic acid. Different types of activated carbon for adsorbing OBS have been systematically investigated, and the results show that the granular-activated carbon activated by potassium hydroxide presents the optimum adsorption capacity of OBS at 219 mg/g depending on the π–π interaction and hydrophobic interaction, which took 70 h to reach adsorption equilibrium [29]. Meanwhile, reduced graphene oxide/Fe_3_O_4_ for the adsorption of OBS were studied simultaneously in 2019, finding that the maximum adsorption capacity was only 362.4 μmol/g within 67 h, the adsorption mechanism of which included hydrophobic, hydrogen, and π–π interactions [22]. These adsorption materials for OBS exhibited the disadvantages of a long adsorption time and trivial adsorption capacity. Therefore, the development of positive adsorbents to facilitate an electrostatic interaction in the OBS terminal anion group might be effective for the adsorption. 

Chitosan as a positively charged compound could promote adsorbing anionic pollutants owing to quantities of free amino groups, but its poor mechanical stability limits a wide application. Graphene oxide (GO) is an important derivative of graphene with decent mechanical stability, and which possesses oxygen-containing groups and π–electron systems. This similar physicochemical property to OBS might improve its removal efficiency. Therefore, GO cross-linked by chitosan was prepared for anion OBS adsorption, which might generate an electrostatic interaction to improve the adsorption capacity of OBS. Moreover, CS-GO possessed the bonds of π, hydrogen, and hydrophobic groups simultaneously. These π–π interactions, hydrogen bonding, and hydrophobic interactions could facilitate the removal of OBS synergistically. 

In order to solve the adsorption of OBS effectively, this study aimed to achieve three objectives: (1) characterizing the synthesized CS-GO composites, compared with graphene oxide (GO) and graphene (GH) simultaneously; (2) investigating single influence factors containing the types of adsorption materials, adsorbent dosage, pH, and inorganic ions of solution; (3) comparing the adsorption kinetics of CS-GO, CS, GO, and GH toward OBS; and (4) revealing the adsorption mechanism of OBS based upon the density functional theory (DFT) at the molecular level.

## 2. Materials and Methods

### 2.1. Chemicals

Analytical reagent-grade sodium p-perfluorinated noneoxybenzen sulfonate (OBS), graphite, graphene, potassium permanganate (KMnO_4_), sodium chloride (NaCl), hydrogen peroxide (H_2_O_2_), calcium chloride hexahydrate (CaCl_2_.6H_2_O), chloride magnesium (MgCl_2_), sodium hydroxide (NaOH), concentrated sulfuric acid (H_2_SO_4_), phosphoric acid (H_3_PO_4_), and concentrated hydrochloric acids (HCl) were purchased from McLean Biochemical Technologies Ltd. (Shanghai, China). HPLC-grade methanol was purchased from Fisher Chemical Company (USA). All solutions used in the experiments were prepared with ultrapure water in an electrical conductivity of 18.2 MΩ/cm (Millipore, Bedford, MA, USA).

### 2.2. Preparation of GO and CS-GO

A 6:1 mixture of potassium permanganate and graphite was added into a beaker containing H_2_SO_4_ and H_3_PO_4_ with a proportion of a 9:1 ratio. The volume of H_2_SO_4_ was 36 mL with a concentration of 95% and H_3_PO_4_ was 4 mL with a concentration of 85%. Heating and stirring were performed for 12 h at 50 °C. The unreacted potassium permanganate was washed away with 30% H_2_O_2_. Subsequently, the above mixture was centrifugated at 5000 rpm within 30 min, and the precipitate was cleaned to neutrality using ethanol, hydrochloric acid, and ultrapure water. Ultimately, the precipitate was dissolved in ultrapure water and vacuum-dried in a freeze-dryer. 

A chitosan solution was prepared by dissolving 3 g of chitosan in 100 mL of 2% acetic acid under magnetic stirring for 10 h. A GO dispersion solution was prepared by dispersing 0.5 g of graphene oxide in 100 mL of deionized water with ultrasonication for 150 min. Subsequently, the chitosan solution and the GO dispersion were mixed in a mass ratio of 1:5, 3:5, and 10:1. The reaction was placed in a hydrothermal reactor at 120 °C for 12 h and then freeze-dried at −60 °C for 40 h, generating the CS-GO (1:5), CS-GO (3:5), and CS-GO (10:1) composite materials, respectively.

### 2.3. Characterization

The surface morphology of materials was characterized by scanning electron microscopy (FEI-NOVANANOSEM 450, FEI Company, Hillsboro, WA, USA). The specific surface area and pore diameter distribution of the materials were measured by an automatic specific surface area tester (Mike ASAP 2460, Micromeritics, Norcross, GA, USA) by using the Brunauer–Emmett–Teller method. Fourier transform infrared spectroscopy (IRPrestige-21, Shimadzu Company, Tokyo, Japan) was used to investigate the element composites and functional groups of materials. X-ray photoelectron spectroscopy (Thermo Scientific K-Alpha, Thermo Scientific company, Waltham, MA, USA) was used to measure the element and chemical composition of the materials’ surfaces.

### 2.4. Adsorption Experiments

Batch adsorption experiments were performed on 100 mL OBS solutions with a concentration of 50 mg/L by adding various absorbents into conical tubes with the track oscillator at a constant temperature and a speed of 150 rpm. The adsorption period was 10 h with a sampling time of every 10, 20, 30, 60, 120, 240, 480, and 600 min. After the optimal CS-GO material was selected, single factor experiments on the OBS adsorption influence involving the adsorbent dosage, solution pH, and inorganic ions were conducted, respectively. The dosage of the adsorbent ranged from 1 mg to 5 mg, the pH ranged from 3 to 11, and the inorganic ions included Na^+^, Mg^2+^, and Ca^2+^. The pH was adjusted by 0.01 mol/L NaOH and HCl. Ultimately, the concentrations of each sample were determined by a UV-vis spectrophotometer after filtration using a 0.45 μm organic filter membrane.

### 2.5. Analysis Method

The wavelength of the sample was scanned using an ultraviolet and visible (UV-vis) spectrophotometer (DR 5000, Hach Company, Loveland, CO, USA). The absorbance was determined to calculate the concentration and removal efficiencies of OBS with maximum absorption wavelengths of 218 nm. The standard curve for OBS was y = 46.6345x + 0.4750, R^2^ = 0.999901.

### 2.6. Data Analysis

#### 2.6.1. OBS Removal Rate E and Adsorption Amount q_e_ for Unit Adsorbent Calculated Formulas


(1) $$E=\frac{C_{0}-C}{C_{0}}$$

E: The removal efficiency of OBS, %;
C_0_: The initial concentration of OBS, mg/L;
C: The concentration of OBS at i-time, mg/L.

(2) $$q_{e}=\frac{{(C}_{0}-Ce)\times V}{m}$$

Ce: The equilibrium concentration of OBS, mg/L;
V: The volume of solution, L;
m: Dosage of adsorbent, mg;
q_e_: The equilibrium adsorption capacity of the adsorbent, g/g.

#### 2.6.2. Adsorption Dynamic Models

Pseudo-first-order kinetics equation:

(3) $$\ln\left(q_{e}-q_{t}\right)=lnq_{e}-k_{1}t$$

q_t_: The adsorption capacity of adsorbent at time t, mg/g;
k_1_: Adsorption rate constant of pseudo-first-order kinetic equation, min^−1^.

Pseudo-second-order kinetics equation:

(4) $$\frac{t}{q_{t}}=\frac{1}{k_{2}{q_{e}}^{2}}+\frac{t}{q_{e}}$$

k_2_: Adsorption rate constant of the pseudo-second-order kinetic equation, [g/(mg⋅min)].

### 2.7. Computational Methods of Quantum Chemistry

Density functional theory (DFT) is effective for predicting the chemical reactivity and interaction mechanism of molecules [30,31,32]. Material studio (MS) was employed to perform a DFT calculation and examine molecular dynamics in this study.

Adsorption energy calculation: Firstly, the model of the adsorbent material and the adsorption system was constructed. Secondly, the charge number of the adsorption system was corrected by the automatic correction function of the force field type. Subsequently, the adsorption system was geometry optimization by the Forcite module with the following parameters: The force field adopted Compass II; the convergence of energy, force, and displacement was 1 × 10^−4^ Kcal/mol, 0.005 Kcal/mol/Å, and 5 × 10^−5^ Å, respectively. Finally, the Forcite module was used to calculate the adsorption energy (Ead) of the material on the contaminant, and the Ead calculation formula was shown in Equation (5).

Molecular dynamics calculation: Firstly, the solution model containing water molecules, OBS, and adsorbent molecules was constructed by the amorphous cell (AC) module, and 20 frames of the solution model were output to select the lowest energy for geometry optimization, with the following parameters: The force field adopted Compass II, and the convergence of energy, force, and displacement was 1 × 10^−4^ Kcal/mol, 0.005 Kcal/mol/Å, and 5 × 10^−5^ Å, respectively. Secondly, molecular dynamics calculation was carried out on the optimized solution model using Forcite molecules with the following parameters: The force field adopted Compass II, with a temperature at 298 K, NVT equilibrium, and a nose for the thermostat. After the calculations were completed, the OBS was selected in the solution model, and the mean square displacement analysis was performed on frames 251–500 of the solution model trajectory file. MSD was used to analyze the molecule dynamics. The specific formula was shown in Equation (6).

(5) $$E_{ad}=E_{total}-E_{a}{-E}_{b}$$

E_ad_: Adsorption energy, Kcal/mol;
E_total_: The energy of adsorption system, Kcal/mol;
E_a_: The energy of adsorbent, Kcal/mol;
E_b_: The energy of OBS, Kcal/mol.

Diffusion coefficient equation

(6) $$D=\frac{1}{6}\underset{t\to \infty }{\lim}\frac{dMSD(t)}{dt}$$

MSD: Mean square displacement, cm^2^/s.

## 3. Results

### 3.1. Characterization of Adsorbents

#### 3.1.1. SEM Analysis

**Figure 1:**
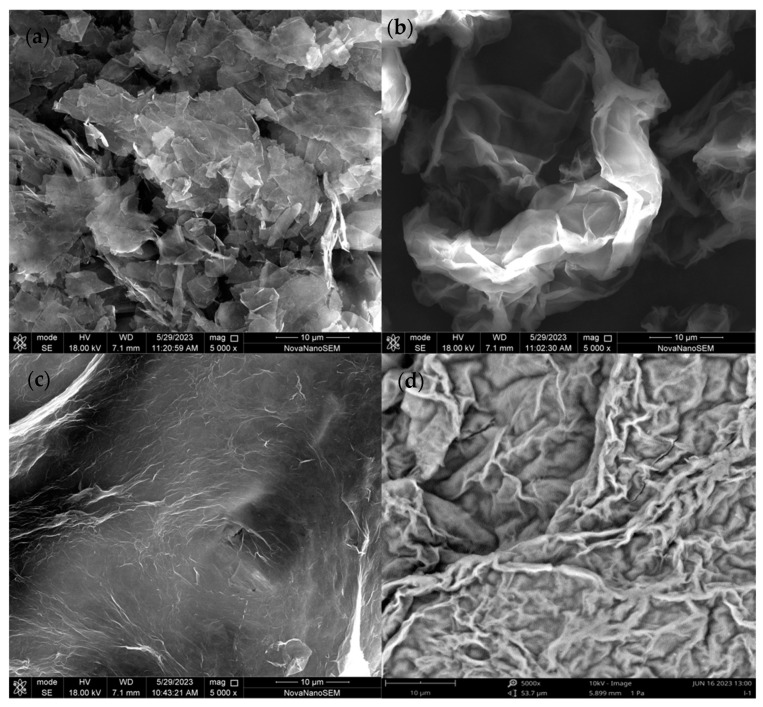
The SEM images of GH (**a**), GO (**b**), CS-GO (1:5) (**c**), and CS-GO (10:1) (**d**).

In this study, the surface morphology and structure of graphene (GH) and graphene oxide (GO), together with chitosan–graphene oxide (1:5 and 10:1), were characterized by SEM. As shown in Figure 1a, GH revealed a multilayered and overlapping structure, which might be related to the π–π interaction between graphene layers [33]. In Figure 1b, GO presented corrugated layers and numerous folds with smooth edges on the surface, and there was no agglomeration between the graphene oxide layer compared with GH, which proved the successful synthesis of graphene oxide. In Figure 1c, CS-GO (1:5) possessed a three-dimensional porous, smooth, and even morphological structure, which proved chitosan was cross-linked on GO successfully. In Figure 1d, CS-GO (10:1) was further uniformly rough and multiply folded, and it was significantly more folded than CS-GO (1:5) due to the supplementary chitosan being cross-linked on the GO surface. 

In addition, the energy dispersive spectrometer (EDS) analysis showed the main element of GH was C and for GO, the main elements were C and O, while CS-GO (1:5) appeared for N, which indicated the CS was synthetized on the GO surface successfully. The information on EDS is shown in Table 1.

#### 3.1.2. BET Analysis

**Figure 2:**
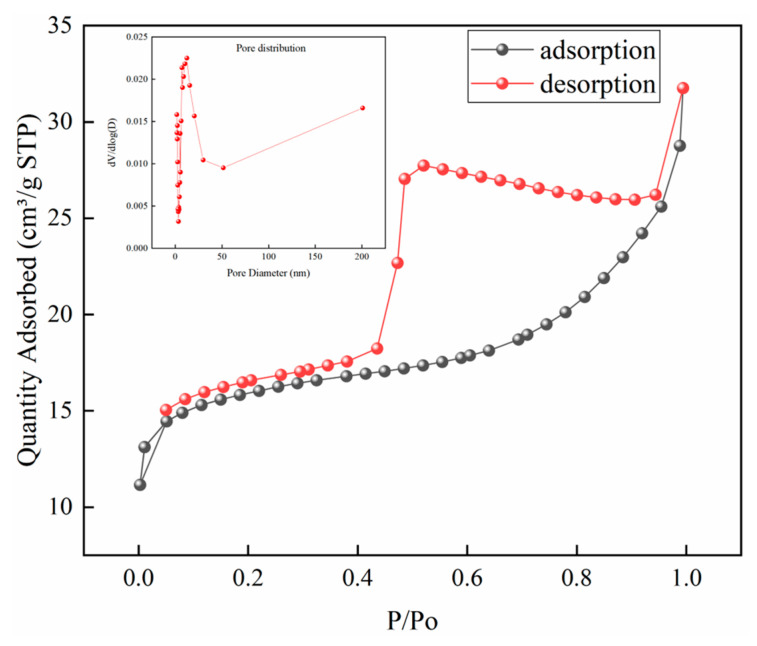
The nitrogen adsorption/desorption isotherm of GO.

BET is important for evaluating the specific surface area and pore structure of materials; thus, nitrogen adsorption/desorption isotherm experiments of GO were conducted under 77 K. The nitrogen adsorption/desorption isotherm curve of GO is shown in Figure 2. Based on the Brunauer–Emmett–Teller method, the specific surface area of GO was calculated to be 58.95 m^2^/g. The isotherm curve of GO shown in Figure 2, which exhibited a typical type-IV curve with an H4 hysteresis loop, indicates that GO presented a mesoporous structure. The inset in Figure 2 showed the pore size distribution curve of GO based on BJH calculation, and it indicated that there were small mesoporous pores (7–12 nm) primarily in GO, and the pore size distribution was not uniform. The pore distribution of CS-GO (10:1) was not measured due to the small specific surface area, which indicated that the physical adsorption capacity of the material was unsatisfactory.

#### 3.1.3. FTIR Analysis

**Figure 3:**
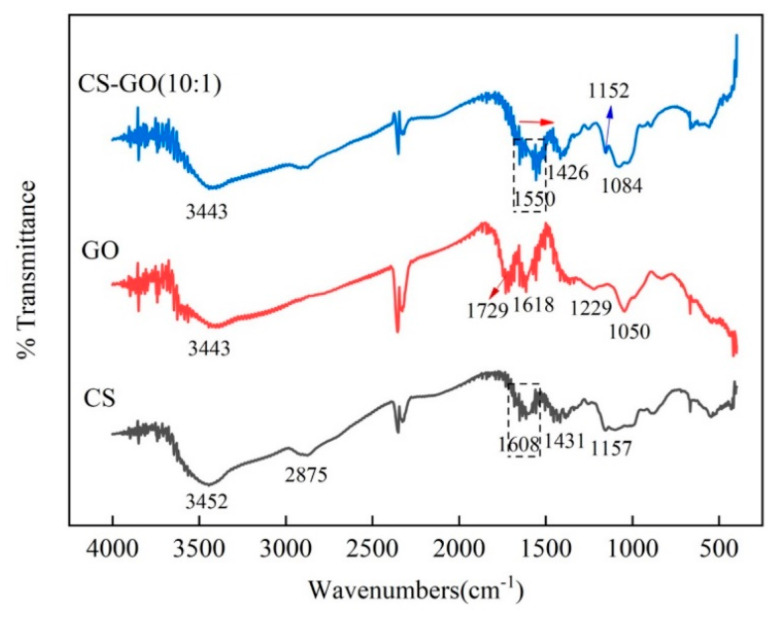
The FTIR spectrum of CS, GO, and CS−GO (10:1).

FTIR analysis of CS, GO, and CS-GO (10:1) is presented in Figure 3. The FTIR spectrum of CS showed the characteristic peaks at 1157 cm^−1^, 1431 cm^−1^, 1608 cm^−1^, 2875 cm^−1^, and 3452 cm^−1^, which corresponded to the stretching vibration of C-O, the bending vibration of -CH_2_, the bending vibration of -NH_2_, and the stretching vibration of C-H and O-H, respectively. The characteristic peaks of GO appearing at 1050 cm^−1^ and 1229 cm^−1^ were assigned to the stretching vibration of C-O-C and C-O, respectively. Another two typical peaks at 1618 cm^−1^ and 1729 cm^−1^ corresponded to the stretching vibrations of C=C and -C=O, respectively. In addition, the peak that appeared at 3443 cm^−1^ was the stretching vibration of O-H. The FTIR spectrum of CS-GO (10:1) showed that the characteristic peak of -NH_2_ of amide groups (-C(=O)-NH_2_) was red-shifted from 1608 cm^−1^ to 1550 cm^−1^, which might be attributed to electrostatic interaction [34], and another characteristic peak presented at 1426 cm^−1^ attributed to the stretching vibrations of C=O in -C(=O)-NH_2_. Moreover, other peaks that presented at 1084, 1152, and 3443 cm^−1^ attributed to stretching vibrations of C-O, C-N, and O-H, respectively. The presence of characteristic peaks of amide groups demonstrated that the CS-GO composite material was synthetized successfully.

### 3.2. Single Influence Factors Studies of Adsorption

#### 3.2.1. Influence of Adsorption Materials

**Figure 4:**
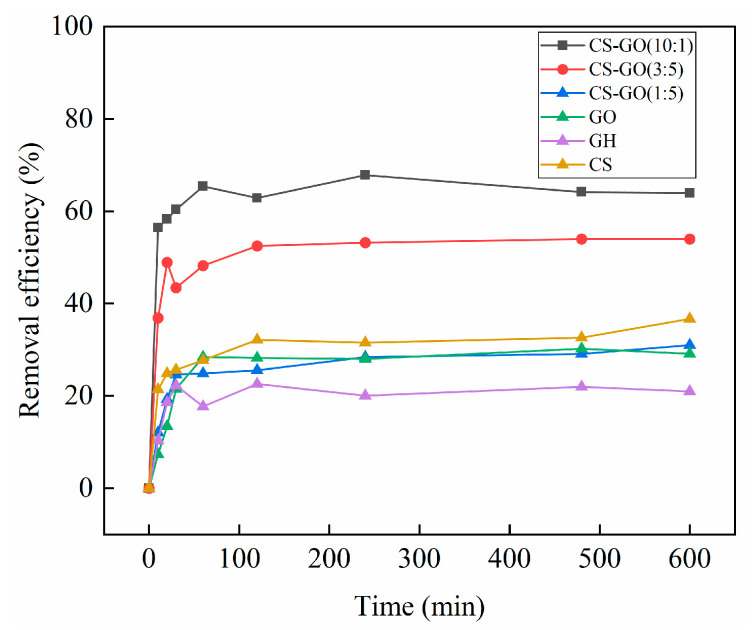
The influence of materials on OBS adsorption. (temperature: 25 °C; initial concentration: 50 mg/L; ratio of sample mass: 10 mg/L; solution volume: 100 mL).

Due to the electron-friendly characteristics of the OBS terminal group, improving the functional group’s affinity between adsorbents and OBS might enhance the OBS removal efficiency. Therefore, diverse functional groups of adsorption materials were investigated for the adsorption capacity of OBS in this study, including CS-GO (10:1), CS-GO (3:5), CS-GO (1:5), GO, and GH. As shown in Figure 4, the OBS removal efficiency of CS-GO (10:1) was significantly superior to CS, GO, and GH. The OBS removal efficiencies of CS-GO (10:1), CS-GO (3:5), CS-GO (1:5), CS, GO, and GH were 60.3%, 43.4%, 24.6%, 25.7%, 21.5%, and 22.3% at 30 min, respectively. This phenomenon showed that the adsorption capacity of material on OBS was gradually improved with the increase in chitosan content, which might be related to the increasing quantities of amino groups in the composite with the addition of CS. In general, the negatively charged groups of -COO or -SO_3_^−^ of PFAS take electronic attraction with the positively charged -NH_3_^+^ [35,36]. Therefore, the electrostatic force between the material and anion OBS would greatly promote the adsorption efficiency. CS-GO (10:1) presented the fastest removal efficiency of 58.3% for OBS in the initial 20 min, and reached an equilibrium within 2 h, with a removal efficiency of 65.4%. This phenomenon might be related to the increased electrostatic interaction due to the positive charge of the material group [28]. However, GH and GO did not present a significant performance due to the absence of electrostatic interaction. The adsorption mechanism of GO depends on hydrogen bonding and π–π interactions, while GH depends on hydrophobic and π–π interactions according to previous studies [37,38,39]. Therefore, the adsorption material was chosen as CS-GO (10:1) in the subsequent study.

**Figure 5:**
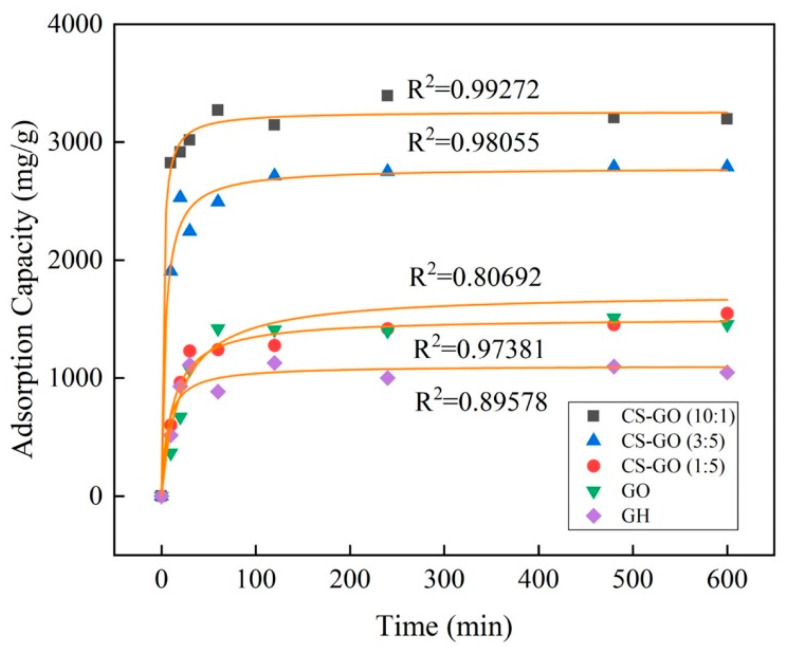
The pseudo-second-order adsorption kinetics of different materials (temperature: 25 °C; initial concentration: 50 mg/L; ratio of sample mass: 10 mg/L; solution volume: 100 mL).

In order to further investigate the control mechanism in the process of OBS adsorption by diverse materials, pseudo-first-order and pseudo-second-order kinetic models were employed to fit the OBS adsorption. As shown in Figure 5, the adsorption capacity of OBS on the five materials involving CS-GO (10:1), CS-GO (3:5), CS-GO (1:5), GO, and GH gradually increased over time, and the adsorption reached equilibrium within 4 h. CS-GO (10:1) exhibited the maximum equilibrium adsorption capacity of 3196.8 mg/g among the five materials. All fitting results are shown in Table 2. The R^2^ of OBS adsorption by the three composite materials of CS-GO from the pseudo-first- and pseudo-second-order kinetics all exceeded 0.95, indicating that both chemical adsorption and physical adsorption were altogether involved in the adsorption process. The pseudo-second-order kinetic model could better fit the OBS adsorption data of the three composite materials, and the regression coefficients R^2^ were 0.9927, 0.9806, and 0.9738, respectively. It was demonstrated that the adsorption process of CS-GO was mainly controlled by chemical adsorption. Moreover, the OBS adsorption data from GO and GH were well-fitted with the pseudo-first-order kinetic model, indicating that the adsorption rate was controlled by the diffusion step in the adsorption process. In addition, according to the k value of the pseudo-second-order kinetics, CS-GO (10:1) presented the maximum adsorption rate constant among the above five materials, followed by CS-GO (3:5) and CS-GO (1:5), and GO expressed the slowest adsorption rate.

#### 3.2.2. Influence of Adsorbent Dosage

**Figure 6:**
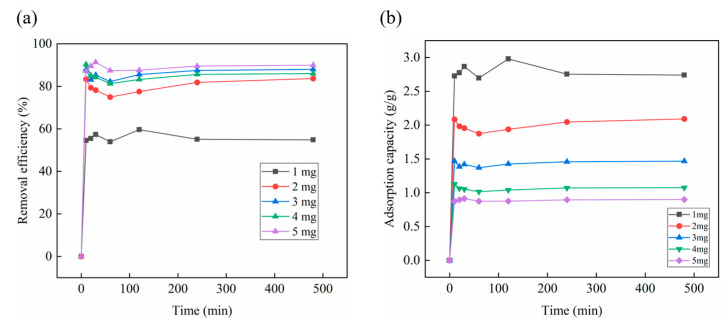
The influence of adsorbent dosages on OBS removal efficiency (**a**) and adsorption capacity (**b**) (temperature: 25 °C; initial concentration: 50 mg/L; solution volume: 100 mL).

The adsorbent dosage is one of the most important influence parameters in the adsorption process. In this study, the dosage of CS-GO (10:1) was adopted as 1, 2, 3, 4, and 5 mg, respectively. As shown in Figure 6a, the removal efficiencies of OBS were improved with the adsorbent dosage increasing, and the optimum removal efficiency was achieved at 90.0% when the adsorbent dosage was 5 mg. Moreover, the removal efficiency of OBS significantly improved from 55.0% to 83.0% when the adsorbent dosage increased from 1 mg to 2 mg, which might be attributed to the further adsorption sites. However, the removal efficiency of OBS increased slowly when the dosage enlarged from 2 mg to 5 mg. As shown in Figure 6b, the adsorption capacity of OBS decreased as the dosage increased. This might be related to a gradual decrease in the mass ratio of OBS to CS-GO (10:1) in the solution, resulting in a decrease in the utilization rate of CS-GO (10:1) per unit mass. In order to control the economic cost, the adsorbent dosage of 2 mg was selected for the following experiments.

#### 3.2.3. Influence of Solution pH

**Figure 7:**
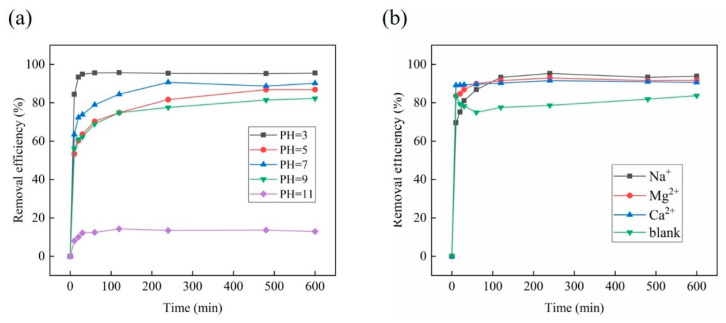
The OBS adsorption influence of solution pH (**a**), and different cations (**b**) (temperature: 25 °C; initial concentration: 50 mg/L; ratio of sample mass: 20 mg/L; solution volume: 100 mL).

pH is a significant influence factor for the adsorption process, which could affect the surface charge of adsorption material, and even the molecular or ionic morphology of contaminants. Theoretically, anion OBS could be adsorbed effectively by positive charge materials. As shown in Figure 7a, it was revealed that CS-GO (10:1) presented a decent removal efficiency of OBS when the pH ranged from 3 to 9, and the optimum removal efficiency was 95.4% when the pH was 3. This was attributed to the amino group of CS-GO (10:1) being protonated, and OBS existed in an anionic form as a negative group of -SO_3_^−^ under acidic conditions [6], which strengthened the electrostatic interaction between them. The removal efficiency of OBS decreased to 13% when the pH increased to 11. This was due to the protonated amino groups on the surface of CS-GO (10:1) translating from positive to negative when the solution environment changed from acidic to strongly alkaline, resulting in the interaction between CS-GO (10:1) and OBS varying from electrostatic attraction to electrostatic repulsion.

#### 3.2.4. Influence of Inorganic Ions

In general, the ions of Na^+^, Mg^2+^, and Ca^2+^ commonly exist in natural waters, and several studies have shown that additional ions could increase or inhibit the adsorption capacity for different adsorbents [40]. Thus, the ions of NaCl, MgCl_2_, and CaCl_2_ with a concentration of 0.01 mol/L were selected to investigate OBS adsorption influence in this study. As shown in Figure 7b, these three cations all intensified the removal efficiency of OBS obviously, and the optimum removal efficiencies were 93.8%, 91.7%, and 90.6% when adding Na^+^, Mg^2+^, and Ca^2+^, respectively. NaCl, MgCl_2_, and CaCl_2_ are electrolytes that can compress the double electric layer of material, which could enhance the affinity of contaminants [41]. In addition, Mg^2+^ and Ca^2+^ could act as a bridge between adsorbents and the negatively charged groups of OBS anions including -OH and -COOH, which could facilitate the adsorptive property of adsorbents [42]. Furthermore, these cations could also be adsorbed on the surface of materials to increase the positive potential, enhancing the electrostatic interaction with anion OBS.

## 4. Adsorption Mechanism

### 4.1. Adsorption Energy

**Figure 8:**
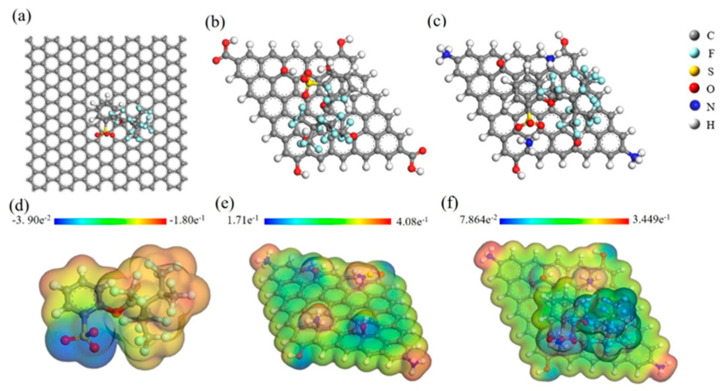
Geometry optimization structure of GH−OBS (**a**), GO−OBS (**b**), CS−GO−OBS (**c**), ESP of OBS (**d**), CS−GO (**e**), and CS−GO−OBS (**f**).

The adsorption mechanism of GH, GO, and CS-GO materials on OBS was investigated using the material studio quantum chemistry computing software system. In order to be more precise, the Forcite tool [32] was employed to conduct geometry optimization and energy calculation of three different adsorption systems including GH-OBS, GO-OBS, and CS-GO-OBS. The adsorption system after geometry optimization is shown in Figure 8a–c. The adsorption energy of GH, GO, and CS-GO on OBS was −96.23 Kcal/mol, −134.10 Kcal/mol, and −227.50 Kcal/mol, respectively. It indicated that the adsorption process was exothermic, while CS-GO presented as extra favorable for adsorption due to maximum adsorption energy. This calculation result was in accordance with the adsorption experiment. The difference in adsorption capacities among the three types of adsorbents might contribute to different functional groups. OBS might be adsorbed on GH by containing the π–π bond., OBS could facilitate the adsorption capacity of GO by a hydrogen bonding interaction generated from hydroxyl functional groups. Compared with the above two adsorbents, CS-GO contained additional functional groups containing -NH_3_, -OH, and π bonds, which could generate electrostatic attraction and hydrogen bonding interactions to enhance adsorption capacity effectively.

Therefore, the electrostatic potential (ESP) of CS-GO before and after adsorption was further investigated using DFT. As shown in Figure 8d, all atoms of OBS were negatively charged with the range from −3.9 × 10^−2^ to −1.8 × 10^−1^. The most active site of OBS was a sulfonic terminal group with a high negative charge, which could cause nucleophilic attacks with adsorbents. Figure 8e showed that the CS-GO surface was positively charged, and the amino groups exhibited the maximum positive charge which attracted OBS effectively. The ESP simulations after adsorption are shown in Figure 8f. For CS-GO-OBS, the ESP still showed a positive charge constantly, but the positive charge density of the amino group decreased due to the neutralization with negative charges of the sulfonic acid group.

### 4.2. Molecular Dynamics

Water molecules might participate in the adsorption process, and therefore their influence in the real environment should be fully considered. In order to further restore the real adsorption system, the solution model was established to study molecular dynamics. Firstly, the AC tool was employed to build a solution model containing water molecules, OBS anions, and adsorbent molecules. Secondly, the configuration with the lowest energy was selected from ten output configurations for structural optimization. Finally, the optimized solution model was calculated for molecular dynamics simulation using the Forcite tool, and the calculation results were analyzed through mean square displacement (MSD). The solution models were nominated as the GH-OBS solution, GO-OBS solution, and CS-GO-OBS solution in this study, which are shown in Figure S1. Moreover, the MSD trajectories of OBS in three different solutions were described in Figure S2 after MSD estimation. Based on Figures S1 and S2, there was correspondence between MSD and the diffusion coefficient of the molecules. The diffusion coefficients of OBS in the GH-OBS solution, GO-OBS solution, and CS-GO-OBS solution were calculated to be 1.20 × 10^−5^ cm^2^/s, 1.60 × 10^−5^ cm^2^/s, and 2.02 × 10^−5^ cm^2^/s, respectively. It is indicated that OBS exhibited the fastest diffusion coefficient in the CS-GO-OBS solution, which was consistent with the fitting results of adsorption kinetics in which CS-GO presented the maximum adsorption rate of OBS.

## 5. Conclusions

In summary, three amino-driven graphene oxide materials of CS-GO with 1:5, 3:5, and 10:1 proportion were developed for the adsorption of OBS in water, compared with GH and GO materials, in this study. Further, the rough uniform, folded, and multilayered appearance shown in SEM, the N element revealed by EDS, and the characteristic peaks of the amide group exhibited from the FTIR spectrum proved that the CS-GO materials were synthesized successfully. Moreover, the adsorption capacity mainly depended on the electrostatic interaction. The optimum removal efficiency of OBS could be achieved up to 95.4% when the adsorbent was selected as CS-GO (10:1), the dosage was 2 mg, and the pH was 3. The addition of Na^+^, Ca^2+^, and Mg^2+^ could altogether enhance the adsorption efficiency of OBS. In addition, based upon the calculation of DFT, MD, and ESP, the adsorption mechanism showed that the sulfonic acid group of OBS was the easiest reactive site, and CS-GO presented extra favorable adsorption due to the maximum adsorption energy and the fastest diffusion coefficient. The electrostatic interaction between the terminal sulfonic acid group of OBS and the amino groups of CS-GO was the foremost motive for improving the adsorption efficiency.

## Figures and Tables

**Table 1:** The EDS information of adsorbents.

Adsorbents	Element	Concentrations	wt%	wt% Sigma
GH	C	75.67	65.98	2.86
	O	1.07	34.02	2.86
GO	C	50.47	98.88	0.90
	O	46.28	1.12	0.90
CS-GO	C	9.94	62.16	6.84
	N	1.25	3.45	7.80
	O	9.74	34.39	5.43

**Table 2:** Kinetic data fitting results of OBS adsorption removal by different adsorbents.

Adsorbents	Kinetics Model	Qe (mg/g)	k	R^2^
CS-GO (10:1)	Pseudo-first-order	3180	0.20 min^−1^	0.98
	Pseudo-second-order	3260	1.74 × 10^−4^ kg/(mg⋅min)	0.99
CS-GO (3:5)	Pseudo-first-order	2663	0.12 min^−1^	0.96
	Pseudo-second-order	2783	8.38 × 10^−5^ kg/(mg⋅min)	0.98
CS-GO (1:5)	Pseudo-first-order	1408	0.058 min^−1^	0.96
	Pseudo-second-order	1509	5.63 × 10^−5^ kg/(mg⋅min)	0.97
GO	Pseudo-first-order	1500	0.038 min^−1^	0.88
	Pseudo-second-order	1720	2.85 × 10^−5^ kg/(mg⋅min)	0.81
GH	Pseudo-first-order	1056	0.088 min^−1^	0.93
	Pseudo-second-order	1106	1.42 × 10^−4^ kg/(mg⋅min)	0.90

## Data Availability

Data are contained within the article.

## Supplementary Materials

The following supporting information can be downloaded at: https://www.mdpi.com/article/10.3390/toxics12050343/s1, Figure S1: Geometry optimization of GH-OBS solution (a), GO-OBS solution (b), and CS-GO-OBS solution (c). Figure S2: MSD trajectories of three different solutions.
