# Supplementary material for: Amino Group-Driven Adsorption of Sodium p-Perfluorous Nonenoxybenzene Sulfonate in Water by the Modified Graphene Oxide
Source: Toxics. 2024 May 8;12(5):343. doi: 10.3390/toxics12050343 (PMC11125578; doi:10.3390/toxics12050343)

Supporting Information

Amino group-driven adsorption of sodium p-perfluorous nonenoxybenzene sulfonate in water by the modified graphene oxide

Mengyuan Lu [1,#], Yang Liu [1,#,*], Xinning Zheng [2], Wenjuan Liu [3], Yang Liu [4], Jia Bao [1,*], Ao Feng [1], Yueyao Bao [1], Jiangyong Diao [5] and Hongyang Liu [5]

[1] School of Environmental and Chemical Engineering, Shenyang University of Technology, Shenyang 110870, China;

[2] Shenyang Zhenxing Sewage Treatment Co., LTD, Shenyang 110143, China;

[3] Dalian Xigang District Center for Disease Control and Prevention, Dalian 116021, China;

[4] Shenyang Hoper Group CO., LTD, Shenyang 110112, China;

[5] Shenyang National Laboratory for Materials Science, Institute of Metal Research, Chinese Academy of Sciences, Shenyang 110016, China

[*] Correspondence: liuyang@sut.edu.cn (Y.L.); baojia@sut.edu.cn (J.B.)

[#] Both authors contributed equally to this paper.

Figure S1. Geometry optimization of GH-OBS solution (a), GO-OBS solution (b), and CS-GO-OBS solution (c)

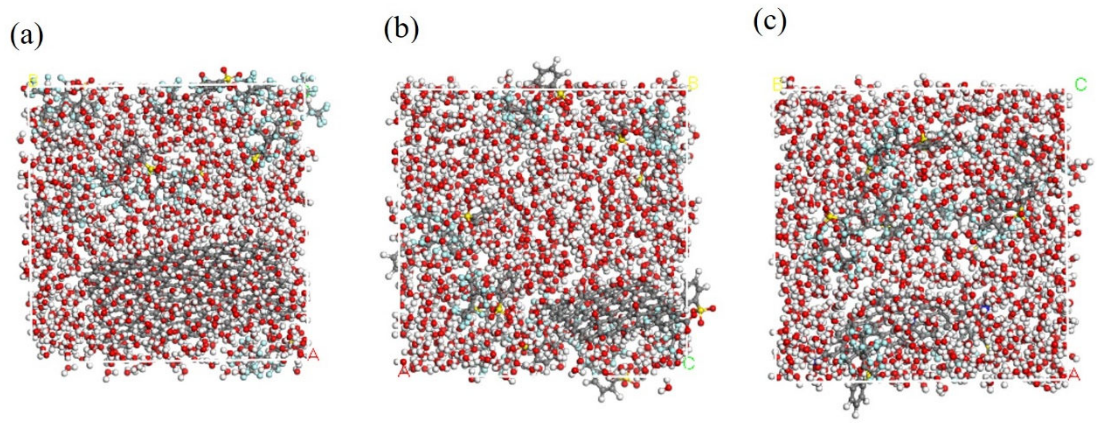

Figure S2. MSD trajectories of three different solutions

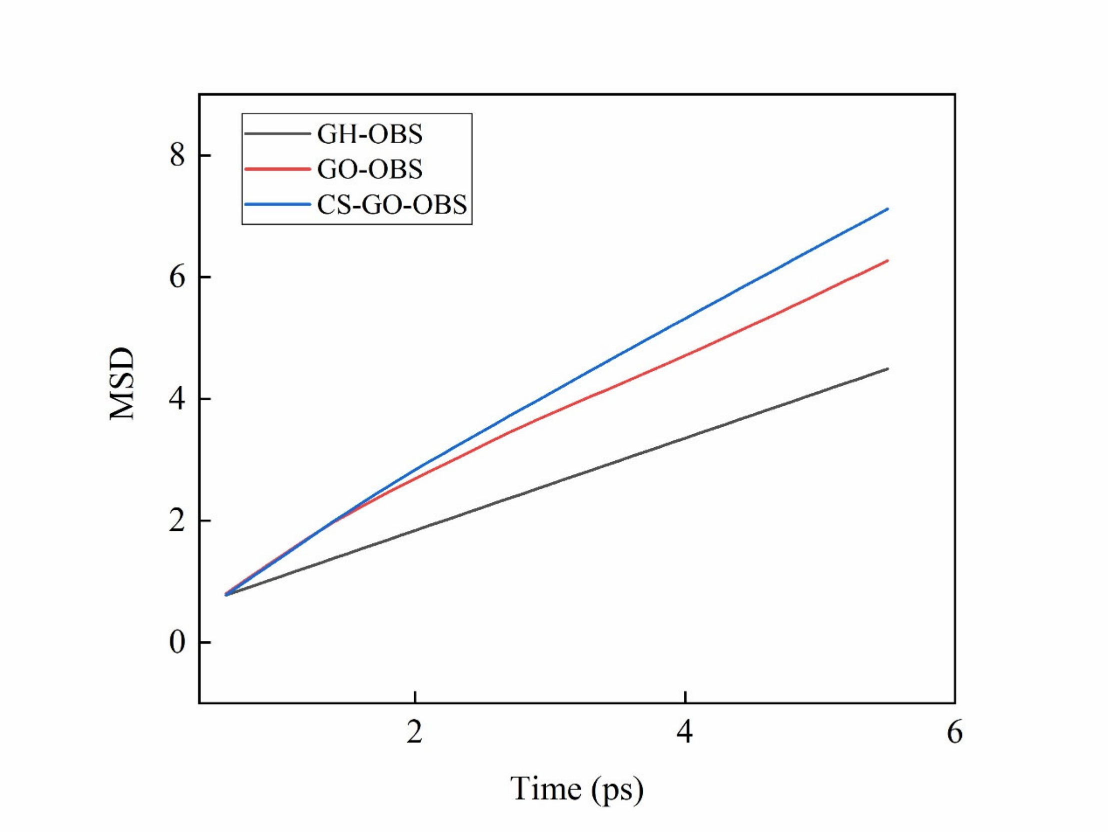

Supplement: Supplementary file 1 [file toxics-12-00343-s001.zip › toxics-2976049-supplementary.pdf]
